# Obituary Notice of the Death of Dr. Hiram Taber

**Published:** 1873-05

**Authors:** 


Obituary. — It is with sincere regret that we announce the death, on May 8th, of Dr. Hiram Taber, of Marilla, in the forty-sixth year of his age. Dr. Taber was a graduate of the Buffalo Medical College in the class of 1852, and has been in the active practice of his profession ever since. He was a gentlemanly, kind and educated physician, beloved by his patients and professional brethren. We have for a long time enjoyed his acquaintance, and shall sadly miss him from our circle of professional friends.

